# How being synanthropic affects the gut bacteriome and mycobiome: comparison of two mouse species with contrasting ecologies

**DOI:** 10.1186/s12866-020-01859-8

**Published:** 2020-07-06

**Authors:** Barbora Bendová, Jaroslav Piálek, Ľudovít Ďureje, Lucie Schmiedová, Dagmar Čížková, Jean-Francois Martin, Jakub Kreisinger

**Affiliations:** 1 Department of Zoology, Faculty of Science, Charles University, Prague, Czech Republic; 2 Studenec Research Facility, Institute of Vertebrate Biology, Czech Academy of Sciences, Brno, Czech Republic; 3 CBGP, Montpellier SupAgro, INRA, CIRAD, IRD, Univ Montpellier, Montferrier-sur-Lez, France

**Keywords:** Microbiome, Metabarcoding, Steppe mouse, Muridae, Symbiosis, Evolution

## Abstract

**Background:**

The vertebrate gastrointestinal tract is colonised by microbiota that have a major effect on the host’s health, physiology and phenotype. Once introduced into captivity, however, the gut microbial composition of free-living individuals can change dramatically. At present, little is known about gut microbial changes associated with adaptation to a synanthropic lifestyle in commensal species, compared with their non-commensal counterparts. Here, we compare the taxonomic composition and diversity of bacterial and fungal communities across three gut sections in synanthropic house mouse (*Mus musculus*) and a closely related non-synanthropic mound-building mouse (*Mus spicilegus*).

**Results:**

Using Illumina sequencing of bacterial 16S rRNA amplicons, we found higher bacterial diversity in *M. spicilegus* and detected 11 bacterial operational taxonomic units with significantly different proportions. Notably, abundance of *Oscillospira,* which is typically higher in lean or outdoor pasturing animals, was more abundant in non-commensal *M. spicilegus*. ITS2-based barcoding revealed low diversity and high uniformity of gut fungi in both species, with the genus *Kazachstania* clearly dominant.

**Conclusions:**

Though differences in gut bacteria observed in the two species can be associated with their close association with humans, changes due to a move from commensalism to captivity would appear to have caused larger shifts in microbiota.

## Background

Various animal species benefit from a commensal association with humans, which offers advantages in terms of extended and more predictable food supplies, lower predation pressure and an ability to spread outside of their original distribution range [1–3]. At the same time, however, commensals may suffer from higher human-induced mortality [4] and a higher infection risk from pathogens specific to humans or other human-associated animals [5]. Moreover, abiotic and biotic factors associated with human-altered and ancestral-habitat environments of human-associated symbionts differ, inducing corresponding changes in selective pressures acting on commensal phenotypes [6]. Consequently, individuals from symbiotic populations may differ morphologically, physiologically and behaviourally from their wild ancestors (e.g. see [7–10]). Despite intensive research in this field, several aspects of phenotypic change due to a commensal lifestyle remain unexplored. In particular, little is known about the effect of a commensal lifestyle on the composition and function of microbial communities harboured within commensal bodies [11] and putative phenotype changes that may be induced by variation in microbial populations.

Animal-associated microbial composition has an important influence on both host health and physiology [12–14], including digestive capacity [15] and interactions with the host’s central nervous [16] and immune systems [17–20] and, as such, can be considered a component of the animal’s phenotype. At the same time, animal-associated microbiota exhibit considerable plasticity due to changing physiological states and environmental conditions, including diet variation, stress or parasite infection [16, 21–23]. Consequently, there is a high expectation that remodulation of a host’s microbiota due to a commensal lifestyle may parallel widely observed changes in microbial populations after translocation of free-living individuals into captivity [24–27].

Murine rodents represent a suitable model group for exploring the effect of commensalism on associated microbiota as the switch between a commensal vs. non-commensal lifestyle has taken place repeatedly during the course of their evolution [1, 28]. To date, however, most research on murine gut microbiota has focused on captive murine species [12, 15, 21, 29–32], commensal populations of murine rodents [33–35] or non-commensal murine species that are phylogenetically distant to commensal taxa [11, 36]. Consequently, there is little comparative data available aimed directly at the assessment of commensalism on microbial structure. This lack of knowledge is even greater as regards eukaryotic microbiota, with the major component in non-commensal animals, the fungal mycobiome, having only been studied exceptionally [37], and never characterised in wild rodents.

To gain an insight into microbial changes due to commensalism in murine rodents, we studied the gut microbiota of two closely related mouse species, the mound-building mouse (*Mus spicilegus*; hereafter MS) and the house mouse (*Mus musculus*; hereafter MM). Together with two other mouse species, MM and MS form a monophyletic group with a genetic distance between them of less than 1% [38]. MS are found in the steppes or in agricultural landscapes and use large mounds built from harvested plants as communal shelters and reproduction sites; importantly, the MS lifecycle is independent of human buildings [39]. Unlike the non-commensal MS, MM benefit from a tight commensal association with humans established ca. 8000 years ago that has enabled its cosmopolitan spread [1]. Consequently, the MM lifecycle is dependent on human infrastructure over most of its current distributional range, including our sample sites.

In our study, we use culture-independent gut microbiota profiling based on high-throughput amplicone sequencing, focusing specifically on the gut bacteriome (hereafter GB), which was characterised using 16S rRNA profiling. Moreover, for the first time, our research describes the gut mycobiome (hereafter GM) structure of a free-living mouse population using ITS2 sequencing. As gut microbial variation in different gut sections from the same individual may exceed interindividual variation of microbial communities sampled within single gut compartments [35], we assessed whether there are any species-specific patterns in gut microbial variation across three gut sections (colon, caecum and ileum).

## Results

### Gut bacteriome

**Fig. 1:**
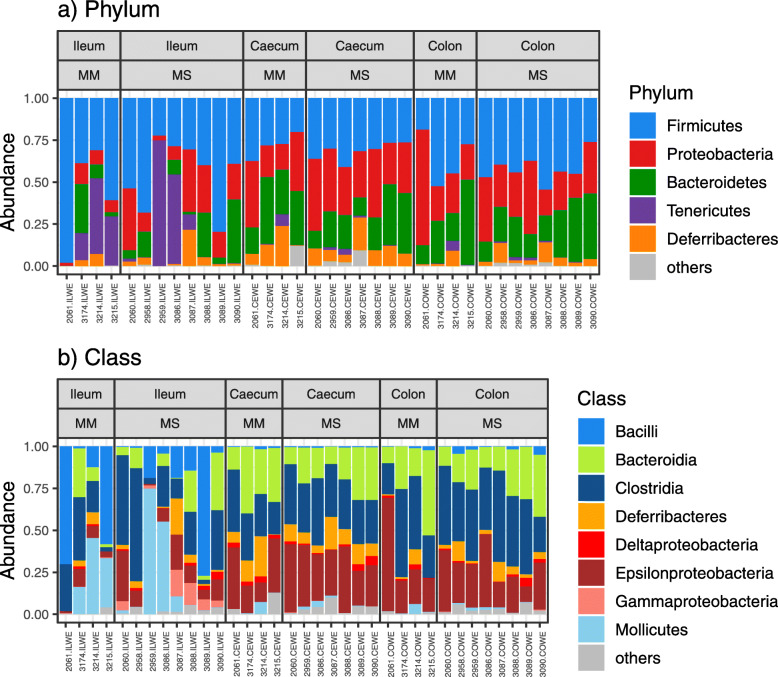
Bar-plot showing the relative abundance of dominant bacterial phyla (**a**) and classes (**b**) in different gut sections (ileum, caecum and colon) and host species (*Mus musculus* – MM; *Mus spicilegus* – MS). Individual bars represent individual samples

GB profiles of the two mouse species were dominated by bacteria of the phyla Firmicutes (40% of all reads), Proteobacteria (25.3%), Bacteroidetes (20%), Tenericutes (7.4%) and Deferribacteres (6.1%, Fig. 1). Another six phyla were detected in our dataset (Spirochaetes, TM7, Actinobacteria, Cyanobacteria, Fusobacteria and Acidobacteria) at low abundances (< 1% reads). At both the phylum and class level, GB content was comparable between MM and MS; however, the dominant bacterial phyla and classes tended to vary between gut sections (Fig. 1, Table S1). In particular, Bacilli (phylum Firmicutes, represented by the genus *Lactobacillus*), Mollicutes (phylum Tenericures, represented by the genus *Mycoplasma*) and Gammaproteobacteria (Proteobacteria, represented by the genus *Aggregatibacter*) were more common in the ileum than the colon or caecum (Fig. 1, Table S1). On the other hand, Bacteroidia (phylum Bacteroidetes, represented by the genera *Bacteroides* and *Odoribacter,* unassigned S24–7 and Rikenellaceae), Clostridia (phylum Firmicutes, represented by unspecified Lachnospiraceae and the genera *Oscillospira* and *Ruminococcus*) and Epsilonproteobacteria (phylum Proteobacteria, represented mainly by the genus *Helicobacter*) were consistently dominant in the colon and caecum.

**Fig. 2:**
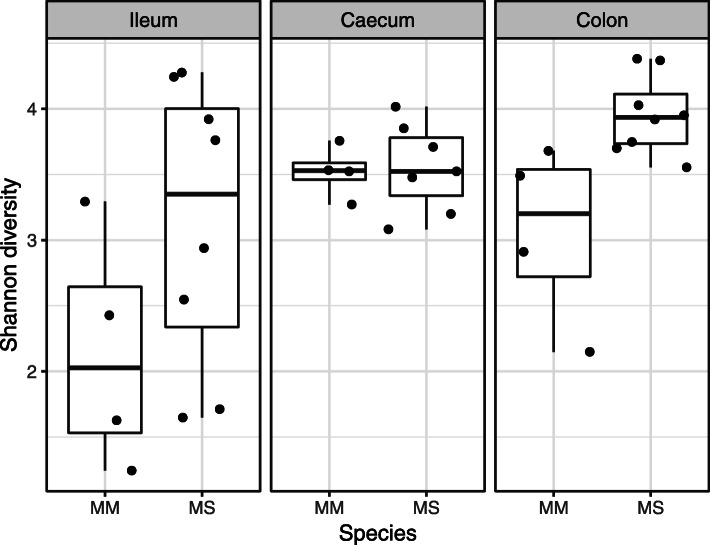
Box-plot showing variation in gut bacteriome Shannon diversity between gut sections (ileum, caecum and colon) and host species (*Mus musculus* – MM; *Mus spicilegus* – MS)

GB alpha diversity varied between gut sections (LME: ΔDF = 2, χ^2^ = 10.62, *p* = 0.005, Table 1, Fig. 2). Specifically, GB was less diverse in the ileum compared to the colon or caecum (Tukey post-hoc comparison: *p* <  0.05 in both cases), whereas the diversity of colon and caecal GB was comparable (Tukey post-hoc comparison: *p* = 0.8395). After the statistical control for this source of variation, GB was more diverse in non-commensal MS compared to commensal MM (LME: ΔDF = 1, χ^2^ = 6.17, *p* = 0.013). At the same time, however, variation in microbial diversity between gut sections showed comparable pattern in the two species, as indicated by nonsignificant species identity vs. gut section interaction (LME: ΔDF = 2, χ^2^ = 3.64, *p* = 0.162). After statistical control for variation between species and gut sections, neither sex (LME: ΔDF = 1, χ^2^ = 0.06, *p* = 0.811) nor sample location (LME: ΔDF = 1, χ^2^ = 0.73, *p* = 0.393) had any effect on GB diversity.

**Table 1:** Minimum adequate model describing the effect of predictors (gut section and species identity) on gut bacteriome alpha diversity variation (Shannon index) in *Mus spicilegus* (MS) and *Mus musculus* (MM). The MS ileal gut bacteriome was used as the reference level (i.e. Intercept) in minimum adequate model parametrization

	Estimate	Std. Error	Df	t value	P value
(Intercept)	2.389	0.260	31	9.187	< 0.001
Gut section (ileum vs. caecum)	0.728	0.290	31	2.508	0.018
Gut section (ileum vs. colon)	0.857	0.284	31	3.017	0.005
Species (MM vs. MS)	0.638	0.248	31	2.575	0.015

**Fig. 3:**
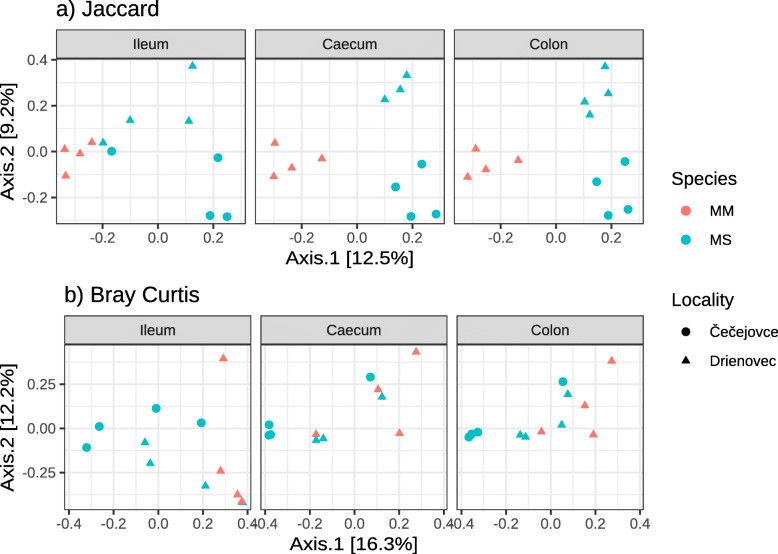
Principal Coordinate Analysis (PCoA) ordination of among-sample divergence for the gut bacteriome of three gut sections (ileum, caecum and colon) from two mouse species (*Mus musculus –* MM; *Mus spicilegus* – MS) and two localities (Čečejovce; Drienovec). Results show the first two PCoA axes running on the Jaccard and Bray-Curtis dissimilarities

According to both OTU prevalence-based (i.e. Jaccard) and relative abundance-based (i.e. Bray-Curtis) dissimilarities, MM vs. MS exhibited divergent GB content associated with the first PCoA axis. In addition, the second PCoA axis tended to separate individuals from different locations. Surprisingly, however, PCoA suggested only slight GB divergence between gut sections (Fig. 3). PERMANOVA analysis indicated that the effect of species and sample location was highly significant for both dissimilarity types, whereas differences between gut sections were only supported by PERMANOVA for relative abundance-based dissimilarity. The effect of sex, despite being marginally significant, explained only < 5% of GB variation (Table 2).

**Table 2:** Results of PERMANOVA analysis testing for the effect of sex, sample location, gut section and species identity on variation in gut bacteriome composition. The Table presents results for absence vs. presence (Jaccard) and relative abundance (Bray-Curtis) dissimilarities not accounting for operational taxonomic unit phylogeny

Dissimilarity	Variable	Df	Sum Of Sqs.	F value	P value	R^2^
Jaccard	sex	1	0.621	1.891	0.004	0.047
	locality	1	1.090	3.320	< 0.001	0.083
	mouse species	1	1.306	3.979	< 0.001	0.099
	gut section	2	0.664	1.012	0.436	0.050
	residual	29	9.520			0.721
Bray-Curtis	sex	1	0.603	2.077	0.007	0.049
	locality	1	1.117	3.851	< 0.001	0.092
	mouse species	1	1.052	3.626	< 0.001	0.086
	gut section	2	0.993	1.711	0.008	0.082
	residual	29	8.414			0.691

**Fig. 4:**
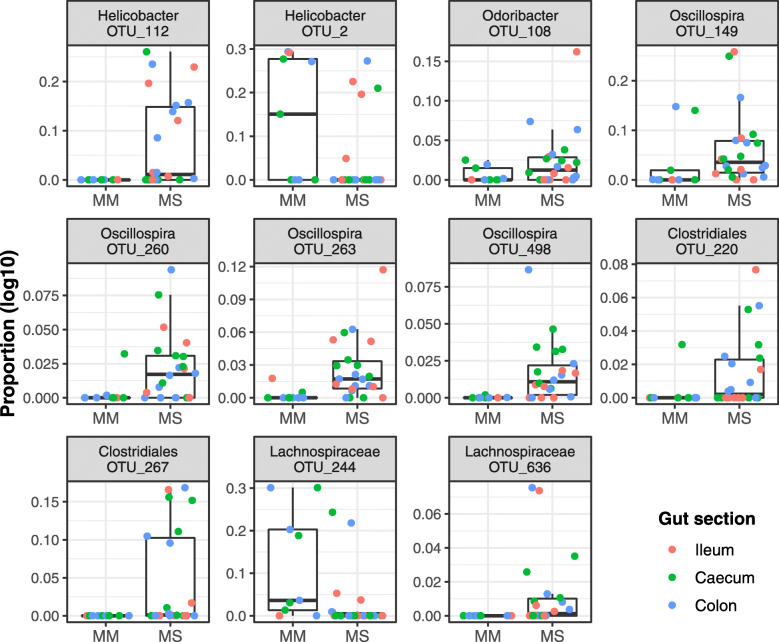
Box-plots for operational taxonomic units (OTU) varying in relative abundance (log_10_ scaled) between *Mus musculus* (MM) and *Mus spicilegus* (MS). Dots = individual observations; gut section indicated by different colours. Genus level assignation for each OTU is provided in each box-plot title

**Fig. 5:**
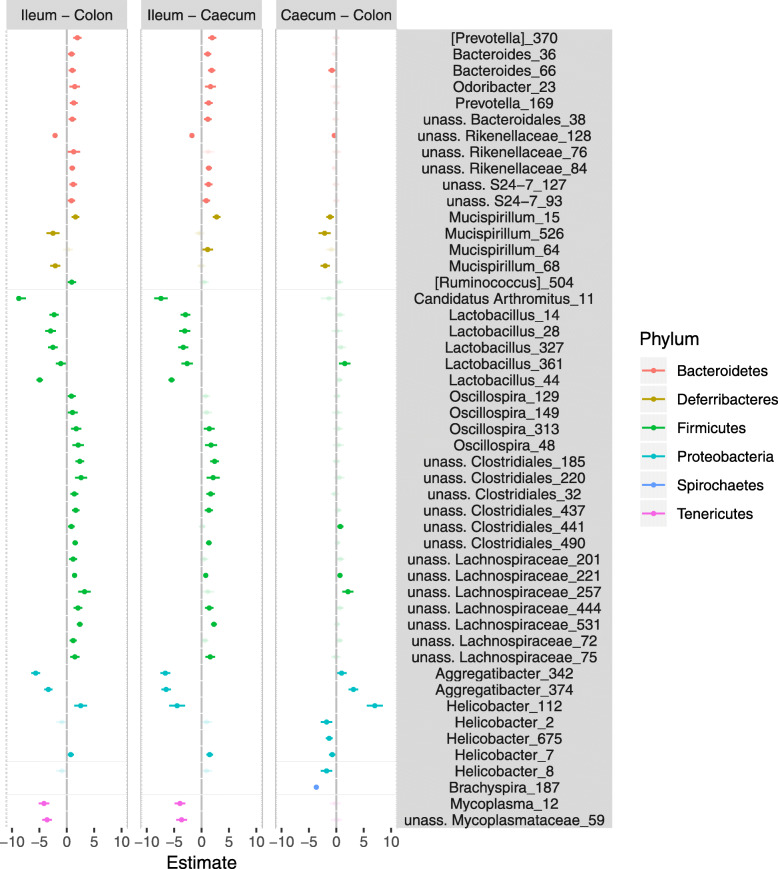
Variation in the abundance of dominant operational taxonomic units between gut sections, with GLMM estimates and 95% confidence intervals for all pair-wise gut section comparisons. Positive values indicate higher abundance in the second named gut section than the first. Non-significant differences based on Tukey comparisons (*p* > 0.05) are indicated by semi-transparent colours. Different colours indicate Phylum level identity

Using negative binomial GLMMs, we detected 11 OTUs (represented by 11% of all reads in our dataset) whose abundances varied between MM and MS. Remarkably, four *Oscillospira*, two OTUs from the family Clostidiaceae and one *Odoribacter* OTU were more abundant in non-commensal MS than commensal MM. The two species also differed in relative abundance of two *Helicobacter* OTUs, one being more abundant in MM and the other in MS (Fig. 4). According to phylogenetic placement analysis, these OTUs represent phylogenetically distant *Helicobacter* species, where *Campylobacter jejuni* (GenBank accession: EU127548.1) is considered as a root (Fig. S1). Furthermore, OTU-level analysis identified 55 OTUs (representing 71% of all reads) whose abundances varied between gut sections (Fig. 5).

### Gut mycobiome

**Fig. 6:**
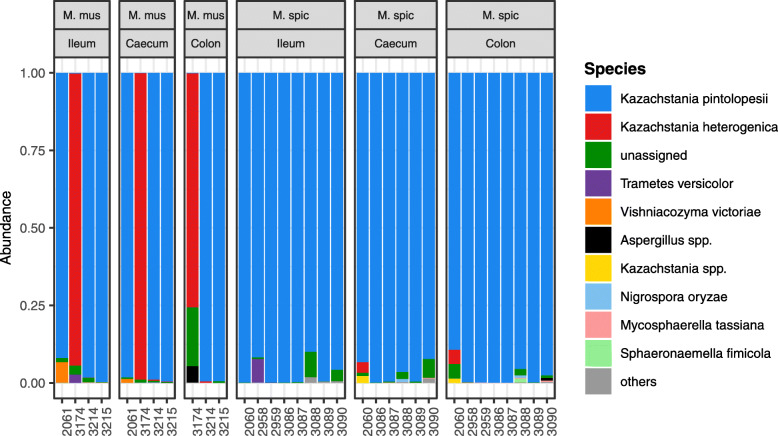
Composition of the murine gut mycobiome. Variation in the dominant fungal taxa of two mouse species (*Mus musculus* and *Mus spicilegus*) and across gut sections. Individual bars represent individual samples

Unlike the GB, GM profiles were highly homogenous across all murine samples, with fungi of the genus *Kazachstania* consistently dominant (representing 97% of all reads and 28 OTUs). According to species-level assignment, most samples comprised *K. pintolopesii*, with the GM of a single MM individual being dominated by *K. heterogenica*. Other fungal taxa were represented by a low proportion of reads (Fig. 6). Alongside the murine samples, we also undertook GM profiling (using the same methodology) of ten faecal microbiota samples from a passerine bird (barn swallow [*Hirundo rustica*], see [40] for details). The GM composition of these samples was much more diverse than the murine GM and covered a broader range of phylogenetically distant fungal taxa. Moreover, *Kazachstania* was almost absent in these non-murine samples (Fig. S2). As such, we conclude that the high homogeneity and low diversity of the murine GM profiles did not arise as an artefact of wet laboratory procedures.

## Discussion

Changes in gut microbiota due to association with humans have been described for a range of vertebrate species [11, 24–27, 34, 41, 42] living in wild vs. in captivity, i.e. zoological gardens [27, 42], domesticated animals [26, 41] and laboratory animals [34, 43]. Overlaying wild vs. captivity microbiome changes for different species potentially allows for the identification of general patterns of microbiome change due to association with humans. On the other hand, the opportunity to establish altered (co)adaptations between microbiota and their hosts in the context of human-altered living environments and lifestyle of the host is limited in captivity. A change in the microbiota of the first generation of animals bred in captivity, for example, may simply represent an unstable, transient state that does not yet show shifts typical for human associated lifestyles. Here, we contrast the gut microbiome of two closely-related mouse species adapted to ecological niches differing greatly in tightness of association with humans (the commensal house mouse [MM] and the non-commensal mound-building mouse [MS]), to get insight on how can commensal association with humans affect bacterial and fungal communities residing in the gut.

In our study, MS displayed a higher gut bacteriome diversity than MM. This may parallel the commonly observed decrease in microbial diversity after introduction of wild animals to captivity [24, 25, 42, 44], ascribed to reduced variation either in environmental factors affecting GB richness (such as diet composition) or in environmental bacteria colonising gut. While GB diversity between the two mouse species showed significant differences, GB diversity variation between different gut sections was even more dramatic, with ileal GB being less diverse than that from the colon and caecum. These within-gut diversity changes are consistent with most previous studies on different mammalian taxa, including rodents [32, 35, 36], and can be explained by spatial gradients in immune function, acidity level, nutrient concentrations and various host-derived secretions within the gut [30, 45, 46].

In addition to changes in GB diversity, we also observed differences in GB composition between the two species. Subsequent differential-abundance analysis identified 11 OTUs (represented by 11% of high-quality reads) whose proportions varied between the two species, with the greatest difference being the increase in abundance of four *Oscillospira* OTUs in MS*.* Interestingly, in parallel to our findings, a previous study on cattle showed that individuals relying on outdoor pasture hosted more abundant *Oscillospira* populations than indoor-bred individuals [47]. According to a few independent studies on human subjects, an increase in *Oscillospira* abundance was associated with reduced body weight [48]. Moreover, a comparative study on several vertebrate species exposed to a fasting regime revealed consistent enrichment of their GB by *Oscillospira* [44]. Together, these observations suggest that variation in the abundance of *Oscillospira* OTUs between MS and MM may reflect differences in nutritional conditions between the commensal and non-commensal niches. Along with *Oscillospira*, two OTUs from the family Clostridiaceae and one *Odoribacter* OTU were more abundant in non-commensal MS than commensal MM. All these taxa (including *Oscillospira*) are anaerobes, capable of fermenting complex plant polysaccharides and producing bioactive short-chained fatty acids (SCFA) with anti-inflammatory capacity [49, 50]. As deficit of SCFA is associated with increased emergence of metabolic disorders and impaired cognitive and immune functions [51], an experimental study linking differences in the abundance of specific SCFA-producing bacteria to potential differences in immune and physiological function between MM and MS would be of great interest., MM and MS gut bacteriome also differed in abundance of two *Helicobacter* and two Lachnospiraceae OTUs. *Helicobacter,* a common inhabitant of murine GB [36, 52], can induce pathological states under certain circumstances [29, 53]. Some *Helicobacter* species are transferred across generations by social contact between community members and, consequently, exhibit phylogeographic codivergence with their hosts [54, 55]. This does not appear to be the case regarding the differences in abundance of *Helicobacter* OTUs in MM and MS, however, as they represent phylogenetically distant lineages and group with *Helicobacter* clades of low host specificity. Particularly, *Helicobacter* OTUs abundant in MS exhibited relatedness to *H. canicola* and *H. bilis*, whereas those abundant in MM clustered with *H. apodemus*.

In this study, considerable differences in GB composition were observed between gut sections, irrespective of host species identity, with the abundance of 55 OTUs (representing 71% of all reads) varying between gut sections. Variation in GB taxonomic content between gut sections was generally congruent with most previously published data on mammalian species [35, 36]. In particular, the distal parts of the gut (i.e. the colon and caecum) were enriched with OTUs of the phylum Bacteroidetes (taxa *Bacteroides*, *Prevotella*, S24–7, Rikenellaceae), class Clostridia (genera *Ruminococcus, Oscillospira* and unassigned Lachnospiraceae) and the genus *Helicobacter* (from phylum Proteobacteria). On the other hand, the ileal GB was characterised by an increased abundance of *Candidatus Arthromitus, Lactobacillus* (both of the phylum Firmicutes), *Mycoplasma* (phylum Tenericutes), *Aggregatibacter* (phylum Proteobacteria) and *Mucispirillum* (phylum Defferibacteres). GB composition also varied between sample locations, implying that the individuals sampled were exposed to different environmental bacterial pools, despite being only 10 km apart, which is consistent with previous studies on wild MM microbiota [33, 43]. Finally, males and females exhibited only slight variation in GB, accounting for ~ 4% of total variation in GB composition.

Surprisingly, both MM and MS exhibited highly uniform gut mycobiome structure, with the genus *Kazachstania* (including two species, *K. pintolopesii* and *K. heterogenica*) as the dominant component, representing ca. 75–100% of high-quality reads. *Kazachstania*, a species complex of Ascomycetous yeasts, has been isolated from a number of captive species, including *K. heterogenica* and *K. pintolopesii* from rodents, *K. slooffiae* from pigs and horses and *K. bovina* from cows and birds [56]. Although little is known about its functions, recent experimental studies have shown the importance of *Kazachstania* for the development of a healthy porcine microbiome, where *Kazachstania* populations support growth of SCFA-producing bacteria [57, 58]. On the other hand, presence of *Kazachstania* in Mongolian gerbils (*Meriones unguiculatus*) exacerbated pathologic effects after experimental infection with *Helicobacter suis* [59]. Similarly, mixed infection of *Kazachstania* and *Escherichia coli* was reported as a causal agent of a fatal disease in captive bred primates [60]. Interestingly, the GM of free-living mice from our populations differed from GM profiles previously reported for captive MM, with captive individuals exhibiting much higher diversity and presence of other prevailing yeast genera, such as *Candida* or *Saccharomyces* [23, 31].

## Conclusions

Massive changes in gut bacteriome composition, explaining ~ 20% of total GB variation, have been documented in several previous studies following introduction of MM from commensal populations to breeding facilities [34]. These changes had a dramatic effect on host physiology, immune function and fitness [43, 61], with important implications on the usage of captive MM colonised by breeding facility-specific gastrointestinal microbiota as models for biomedical research. Similarly, the gut mycobiome profiles reported from captive MM appear to show pronounced differences to the commensal population GM described in this study [23, 31]. The fact that such massive changes in GB and associated host phenotype occurred between two human-associated lifestyles (commensal and captive) leads to the obvious assumption that transition from a wild-living to a commensal niche will have an even more dramatic effect. Nevertheless, our study comparing gut microbiota differences between commensal MM and non-commensal MS suggests relatively low differences in GB content and diversity between the two species, accounting for < 10% of total GB variation. Moreover, mouse GM was surprisingly uniform and consistently dominated by *Kazachstania*, both in commensal MM and non-commensal MS. Consequently, we suggest that translocation from a commensal population to captivity has a comparatively higher effect on mouse gut microbiota than gut microbiota changes associated with adaptation to a commensal niche. Further observation research (1) covering more commensal vs. non-commensal species pairs or (2) focusing on temporal dynamics of gut microbiota changes as well as (3) experimental studies aimed at direct microbiota manipulations would help to uncover details behind variation in gut microbiota vs. host physiology associated with commensal life style.

## Methods

### Sample collection

The mice used in this study were live-trapped in east Slovakia between the 7th and 9th of December 2015, with 4 individuals of MM trapped in a farm granary at Drienovec (N 48° 36.683′, E 20° 56.333′) and 8 individuals of MS obtained from field habitat near Drienovec and Čečejovce (N 48° 33.881′, E 21° 4.137′). In each case, the traps were controlled twice per day, with trapped individuals taken back to the laboratory and caged separately in clean cages with sterile bedding to avoid microbial cross-infection. All mice were sacrificed by cervical dislocation and dissected within 12-h of trapping. Each of the three gut sections (ileum, caecum and colon) was placed on a sterile Petri dish. They were cut longitudinally. The content was gently washed out with sterile physiological saline solution and the tissue samples were placed separately into sterile cryotubes, rapidly frozen in liquid nitrogen and stored at − 80 °C. Whole compartments were taken from the caecum and colon, and the distal part only from the ileum (1 cm). Sampled species are not under legislative protection. Ethical statement regarding sample collection and experimental procedures is provided in “Declarations” section.

### Gut bacteriome genotyping

Metagenomic DNA from gut tissue samples was extracted in April 2016 in a laminar flow cabinet using the PowerSoil DNA isolation kit (MO BIO Laboratories Inc., USA). We failed to collect a caecum sample from one MS individual; consequently, the final dataset included 35 samples of metagenomic DNA (Table S2).

Primers flanking the V3–V4 variable region on bacterial 16S rRNA gene (i.e., S-D-Bact-0341-b-S-17 [CCTACGGGNGGCWGCAG] and S-D-Bact-0785-a-A-21 [GACTACHVGGGTATCTAATCC]) were used during the polymerase chain reaction (PCR) step [62], both forward and reverse primers being tagged with 10-bp barcodes for sample demultiplexing. For the PCR, we used 8.2 μl of KAPA HIFI Hot Start Ready Mix (Kapa Biosystems, USA), 0.56 μM of each primer and 6.2 μl of DNA template. PCR conditions were as follows: initial denaturation at 98 °C for 5 min, followed by 30 cycles each of 98 °C (15 s), 55 °C (20 s) and 72 °C (40 s) and a final extension at 72 °C (5 min). The PCR products, together with negative controls (PCR products for blank DNA isolates), were run on 1.5% agarose gel, the concentration of the PCR product being assessed based on gel band intensity using GenoSoft software (VWR International, Belgium). The samples were subsequently pooled at equimolar concentration and run on 1.5% agarose gel, with bands of appropriate size excised from the gel and purified using the High Pure PCR product Purification Kit (Roche, Switzerland), according to the manufacturer’s instructions. Sequencing adaptors were ligated using TruSeq nano DNA library preparation kits (Illumina, USA) and the resulting amplicon libraries sequenced on a single MiSeq run (Illumina, USA) using v3 chemistry and 2 × 300 bp paired-end reads at Centre de Biologie pour la Gestion des Populations (CBGP, Montferrier sur Lez Cedex, France). Technical PCR duplicates were sequenced for individual samples.

### Gut mycobiome genotyping

The ITS2 region was amplified using universal ITS3 (GCATCGATGAAGAACGCAGC) and ITS4 (TCCTCCGCTTATTGATATGC) primers [63] flanked by oligonucleotides compatible with Nextera adaptors (Illumina, USA). For the PCR reaction, we used 5 μl KAPA HIFI Hot Start Ready Mix (Kapa Biosystems, USA), 0.2 μM of each primer and 4.6 μl of DNA template. PCR conditions were as follows: initial denaturation at 95 °C for 3 min, followed by 30 cycles each of 95 °C (30 s), 53 °C (30 s) and 72 °C (30 s) and a final extension at 72 °C (5 min). Nextera sequencing adaptors were appended to the resulting PCR products during the second PCR round using 10 μl of KAPA HIFI Hot Start Ready Mix (Kapa Biosystems, USA), 2 μM of each primer and 6 μl of PCR product from the first PCR. PCR conditions were as follows: initial denaturation at 95 °C for 3 min, followed by 15 cycles each of 95 °C (30 s), 55 °C (30 s) and 72 °C (30 s) and a final extension at 72 °C (5 min). PCR products of the second PCR were run on 1.5% agarose gel and pooled at equimolar concentration. The final library was cleaned up using Agencourt AmpureXP beads (Beckman Coulter Life Sciences). Products of the desired size (250–700 bp) were extracted by PipinPrep (Sage Science Inc., USA) and sequenced on Illumina Miseq (v3 kit, 300 bp paired-end reads) at the Central European Institute of Technology (CEITEC), Masaryk University, Brno (Czech Republic). Technical PCR duplicates were sequenced for individual samples as in the GB analysis. We failed to amplify and sequence ITS2 in two DNA samples.

### Bioinformatics analysis

Skewer [64] was used for both sample demultiplexing and detection and trimming of gene-specific primers. In the next step, reads of low quality (expected error rate per paired-end read > 1) were eliminated. Dada2 [65] was used for denoising of quality-filtered reads and quantification of 16S rRNA and ITS2 haplotypes (hereafter operational taxonomic units; OTUs) in each sample. Next, UCHIME [66] was employed for detection of chimeric OTUs. The gold.fna (available at: https://drive5.com/uchime/gold.fa) and UNITE databases [67] were used as references for chimeras filtering from the GB and GM datasets, respectively. After elimination of chimeric haplotypes, taxonomy for non-chimeric OTUs was assigned at 80% posterior confidence by RDP Classifier [68]. The GreenGenes database version gg.13.8 [69] was used for bacterial OTU annotation, and the UNITE database [67] for fungal OTU annotation. Bacterial OTU sequences were aligned using PyNast [70] and their phylogenetic tree constructed using FastTree [71]. We did not conduct phylogenetic reconstruction for ITS2 OTUs as ITS2 evolution is driven to a large extent by insertions and deletions, which complicates identification of homologous positions for phylogenetically disparate taxa. OTU tables (i.e. OTU read counts in individual samples), OTU sequences, their taxonomic annotations and phylogeny along with sample metadata were merged into separate GB and GM databases using the package phyloseq [72] in R (R Core Team 2015).

### Statistical analysis

The GM database comprised 589,406 high-quality sequences grouped in 153 non-chimeric OTUs. The number of ITS2 reads per sample ranged between 1826 and 32,002 (median = 17,896). Only basic descriptive tools were used in GM analysis as the sample structure was highly homogenous (detailed below). The GB database comprised 1,088,282 high quality sequences grouped in 1634 non-chimeric OTUs. As the number of bacterial reads varied between samples (range = 1415 – 58,106, median = 29,188), we rarefied the OTU table to achieve even sequencing depth per sample and used the down-sampled database for further statistical analysis unless otherwise stated. The Shannon index was used as an alpha diversity measure, with per-sample Shannon diversities included as a response variable in generalised linear mixed effect models (GLMM), while sex, sampling location, gut section and species identity were included as alpha diversity predictors. We also tested for potential effects of species vs. gut section interaction. Individual identity was included a random term in order to account for pseudo-replication as multiple gut sections were analysed for each individual. Nonsignificant predictors were eliminated from the initial model in a step-wise manner in order to obtain the minimal adequate model [73]. Marginal effects for significant predictors (i.e. effect of predictor in questions controlled for the effects of other significant predictors) are reported. Dissimilarity based on OTU prevalence (binary Jaccard index) and relative abundance (Bray-Curtis index) was used to study divergence in microbiota composition between samples. First, we visualised the between-sample divergence pattern using Principal Coordinate Analysis (PCoA), then applied PERMANOVA (adonis2 function from the vegan R package) to test whether predictors already included in the alpha diversity analysis drove variation in microbial profile composition. To account for within-individual covariance, individual identity was included as a constraint for permutations (i.e. strata). We reported marginal probability values, i.e. significance of the variable in question controlled for the effect of all other variables included in the PERMANOVA model. To identify OTUs exhibiting variation in abundance between different gut sections and between the two species, we employed mixed models (from R package BhGLM [74]) assuming negative binomial error distribution. These analyses were only conducted for a subset of dominant OTUs (represented by > 0.1% reads and present in > 5 samples, *n* = 119). The marginal effect of the predictor in question (i.e. effect of species identity statistically controlled for systematic variation between gut sections and vice versa) was tested using likelihood-ratio tests, with qvalue [75] used as a multiple testing correction method. As in the previous analyses, individual identity was included as a random effect. Tukey post-hoc tests were employed after likelihood ratio testing to identify specific gut section pairs where the OTU in question exhibited significant variation in abundance. In specific cases, phylogenetic placement was used to provide a more detailed insight into OTU taxonomy. A set of reference 16S rRNA sequences exhibiting > 97% similarity with the OTUs in question was extracted from the Silva database v. 132 [76]. Sequences were aligned using mafft [77] and a phylogenetic tree constructed using RAxML [78]. Bootstrap analysis based on 1000 replications was used to estimate support for the tree nodes.

## Supplementary information

**Additional file 1: Figure S1.** Phylogenetic placement of two *Helicobacter* operational taxonomic units whose abundances varied between *Mus musculus* (MM) and *Mus spicilegus* (MS). The tree of partial 16S rRNA sequences was constructed using maximum likelihood. Bootstrap values are based on 1000 replicates. The scale bar represents a 0.04 (4%) nucleotide sequence difference.

**Additional file 2: Figure S2.** Composition of the murine gut mycobiome. Proportion of dominant classes in mouse-mycobiome samples and bird-mycobiome samples (ten barn swallows [*Hirundo rustica*]; see Kreisinger et al. 2017 [40] for details) sequenced in parallel with the mouse samples.

**Additional file 3: Table S1.** Average proportions of bacterial taxa detected in three gut sections (ileum, caecum and colon) of two wild mouse species (*Mus musculus*, *Mus spicilegus*).

**Additional file 4: Table S2.** Sample metadata and sequencing data accession numbers.

## Data Availability

Sequencing data associated with this project are archived in European Nucleotide Archive (project accession numbers: PRJEB37682 and PRJEB37678). Accession numbers for each sample are available in Table S2.

## Abbreviations

MM: *Mus musculus*
MS: *Mus spicilegus*
GB: Gut bacteriome
GM: Gut mycobiome
LME: Linear mixed effect models
PCoA: Principal Coordinate Analysis
OTU: Operational taxonomic unit
PCR: Polymerase chain reaction
CEITEC: Central European Institute of Technology
