# Supplementary figures and images for: How being synanthropic affects the gut bacteriome and mycobiome: comparison of two mouse species with contrasting ecologies
Source: BMC Microbiol. 2020 Jul 6;20:194. doi: 10.1186/s12866-020-01859-8 (PMC7336484; doi:10.1186/s12866-020-01859-8)

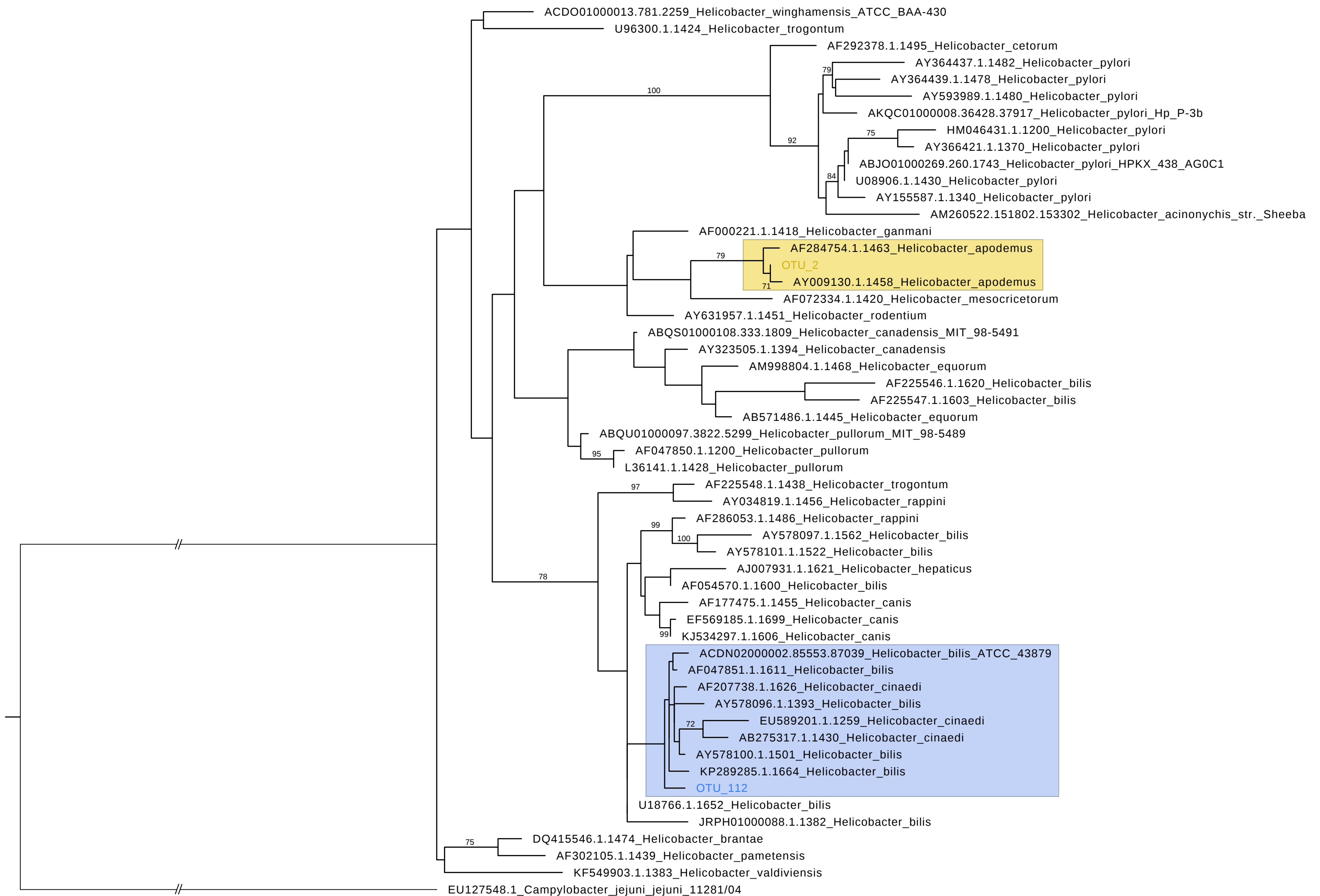

Supplement: Supplementary file 1 — Additional file 1: Figure S1. Phylogenetic placement of two Helicobacter operational taxonomic units whose abundances varied between Mus musculus (MM) and Mus spicilegus (MS). The tree of partial 16S rRNA sequences was constructed using maximum likelihood. Bootstrap values are based on 1000 replicates. The scale bar represents a 0.04 (4%) nucleotide sequence difference. [file 12866_2020_1859_MOESM1_ESM.pdf]

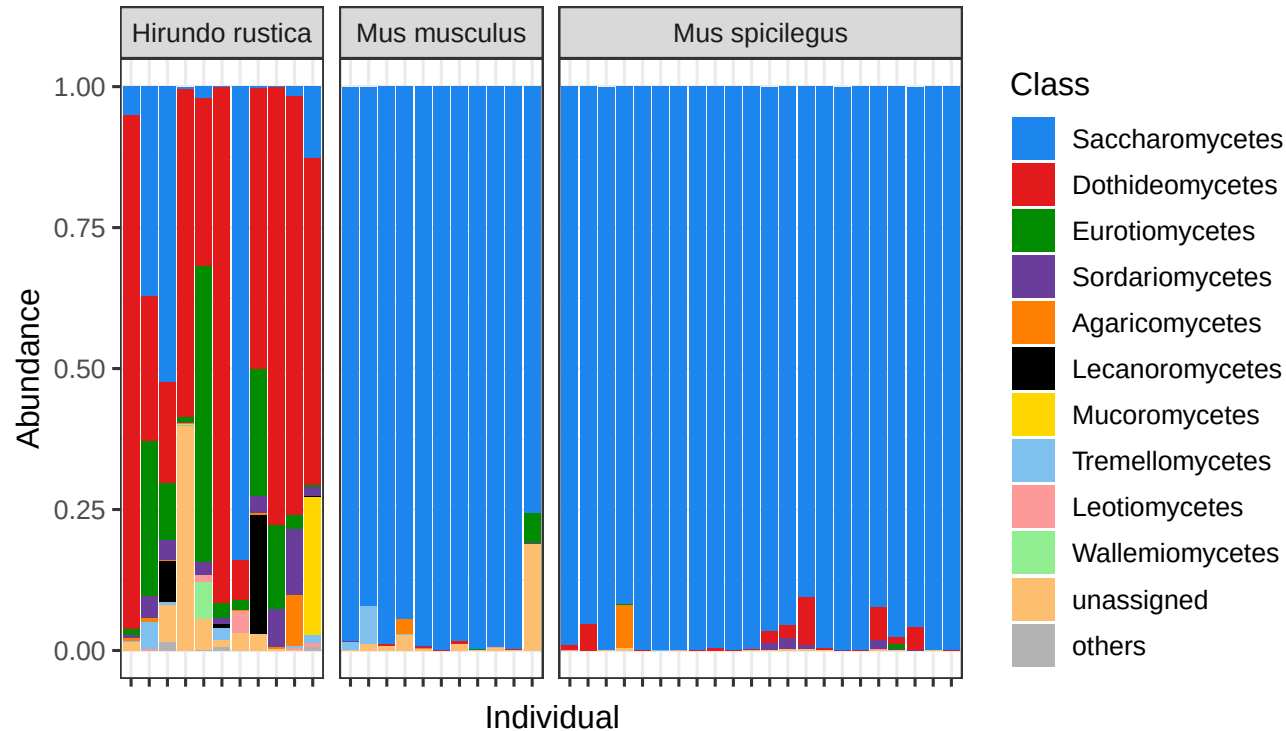

Supplement: Supplementary file 2 — Additional file 2: Figure S2. Composition of the murine gut mycobiome. Proportion of dominant classes in mouse-mycobiome samples and bird-mycobiome samples (ten barn swallows [Hirundo rustica]; see Kreisinger et al. 2017 [40] for details) sequenced in parallel with the mouse samples. [file 12866_2020_1859_MOESM2_ESM.pdf]
